# Association of the blood glucose-to-platelet ratio with all-cause mortality in diabetic patients: An analysis of the MIMIC-IV database

**DOI:** 10.1097/MD.0000000000050128

**Published:** 2026-08-07

**Authors:** Yinan Zhao, Faying Li, Guoying Yu

**Affiliations:** a Clinical Medical College of Qinghai University, Qinghai, China; b Department of Hepatology II, Fourth People’s Hospital of Qinghai Province, Qinghai, China.

**Keywords:** all-cause mortality, blood glucose-to-platelet ratio, critically ill, diabetes mellitus, MIMIC-IV database

## Abstract

Diabetes mellitus is a chronic disease that significantly affects global mortality rates, particularly among critically ill individuals. While previous studies have highlighted the role of glucose regulation and platelet activity in mortality risk, the combined impact of blood glucose and platelet levels remains underexplored. This study aims to investigate the relationship between the blood glucose-to-platelet ratio (BPR) and all-cause mortality in critically ill diabetic patients. A retrospective analysis was conducted using the MIMIC-IV database, which included 21,145 critically ill diabetic patients admitted to the intensive care unit. Patients were categorized into quartiles based on their BPR. Mortality rates at 28 and 360 days post-admission were assessed. Cox proportional hazards models were used to evaluate the association between BPR and hospital mortality, adjusting for potential confounders such as age, sex, comorbidities, and illness severity scores. Restricted cubic spline analysis was applied to assess the nonlinear relationship between BPR and hospital mortality. The median age of patients was 69 years (interquartile range, 60–78), with 58.41% male. The crude 28-day and 360-day hospital mortality rates were 10.83% and 15.57%, respectively. Higher BPR quartiles were associated with increased mortality rates at both 28 days and 360 days (*P* < .001). The hazard ratio for 28-day hospital mortality between the highest (Q4) and lowest (Q1) quartiles was 1.69 in the unadjusted model and 1.36 after adjusting for confounders. Analysis using restricted cubic splines suggested a nonlinear relationship between BPR and hospital mortality, with an increase in risk observed as BPR levels increased. Subgroup analyses revealed consistent associations across different patient demographics and comorbid conditions, including age, sex, and sepsis. BPR appears to be a potential marker for predicting all-cause mortality in critically ill diabetic patients, with a moderate association between BPR and mortality risk. While the blood glucose-to-platelet ratio could serve as a useful tool for identifying high-risk patients, its clinical applicability in intensive care unit settings requires further investigation. Future studies should explore its role in dynamic monitoring and therapeutic interventions, as well as address the limitations of our current findings, such as the observational nature of the data and potential confounding factors.

## 1. Introduction

Diabetes mellitus is a prevalent chronic disease worldwide, and its incidence continues to rise steadily.^[1]^ The World Health Organization estimates that by 2024, approximately 830 million adults globally will be living with diabetes.^[2]^ This condition, characterized by the body’s inability to effectively regulate blood glucose levels, poses an increasingly significant challenge to healthcare systems.^[3,4]^ When poorly managed, diabetes can lead to a range of severe complications, which significantly diminish an individual’s quality of life.^[5]^ Diabetes is primarily classified into 2 types: type 1, which generally manifests during childhood or adolescence, and type 2, which is more common and largely influenced by lifestyle factors such as obesity and physical inactivity.^[6-8]^ Both types are associated with a wide spectrum of complications, including cardiovascular disease, renal failure, diabetic neuropathy, and cerebrovascular events, all of which contribute substantially to the elevated mortality rates observed in individuals with diabetes.^[9,10]^ As the prevalence of diabetes continues to grow, understanding its impact on overall mortality and health outcomes becomes crucial.

The regulation of blood glucose levels is central to the effective management of diabetes and the prevention of its complications.^[11]^ Chronic hyperglycemia is closely linked to an increased risk of severe health conditions, including cardiovascular diseases, neuropathy, and nephropathy.^[11]^ Research has shown a direct correlation between elevated blood glucose levels and the risk of these complications, which can ultimately lead to premature mortality.^[12,13]^ In addition to hyperglycemia, platelet function plays a critical role in diabetes-related complications.^[14]^ In diabetic individuals, platelets often exhibit hyperactivity, which increases the likelihood of thrombotic events and cardiovascular incidents, such as myocardial infarctions and strokes.^[15,16]^ The interaction between blood glucose levels and platelet activity has been explored in various studies, which suggest that both factors significantly contribute to mortality risk in diabetic patients.^[17,18]^ A promising area of investigation is the blood glucose-to-platelet ratio (BPR), which may serve as a simple yet effective metric for evaluating the combined impact of glucose regulation and platelet function on mortality risk. This ratio could offer valuable insights into the systemic effects of diabetes, potentially informing more targeted strategies for mitigating these risks.

This research seeks to assess the prognostic value of the BPR in forecasting all-cause mortality among critically ill diabetic patients, emphasizing the critical need to comprehend diabetes and its associated complications. Utilizing data from the Medical Information Mart for Intensive Care IV (MIMIC-IV), this study investigates the BPR as a potential indicator of mortality risk in diabetic individuals. The objective is to contribute to the development of personalized and proactive healthcare strategies for high-risk populations.

## 2. Materials and methods

### 2.1. Study population

**Figure 1.**
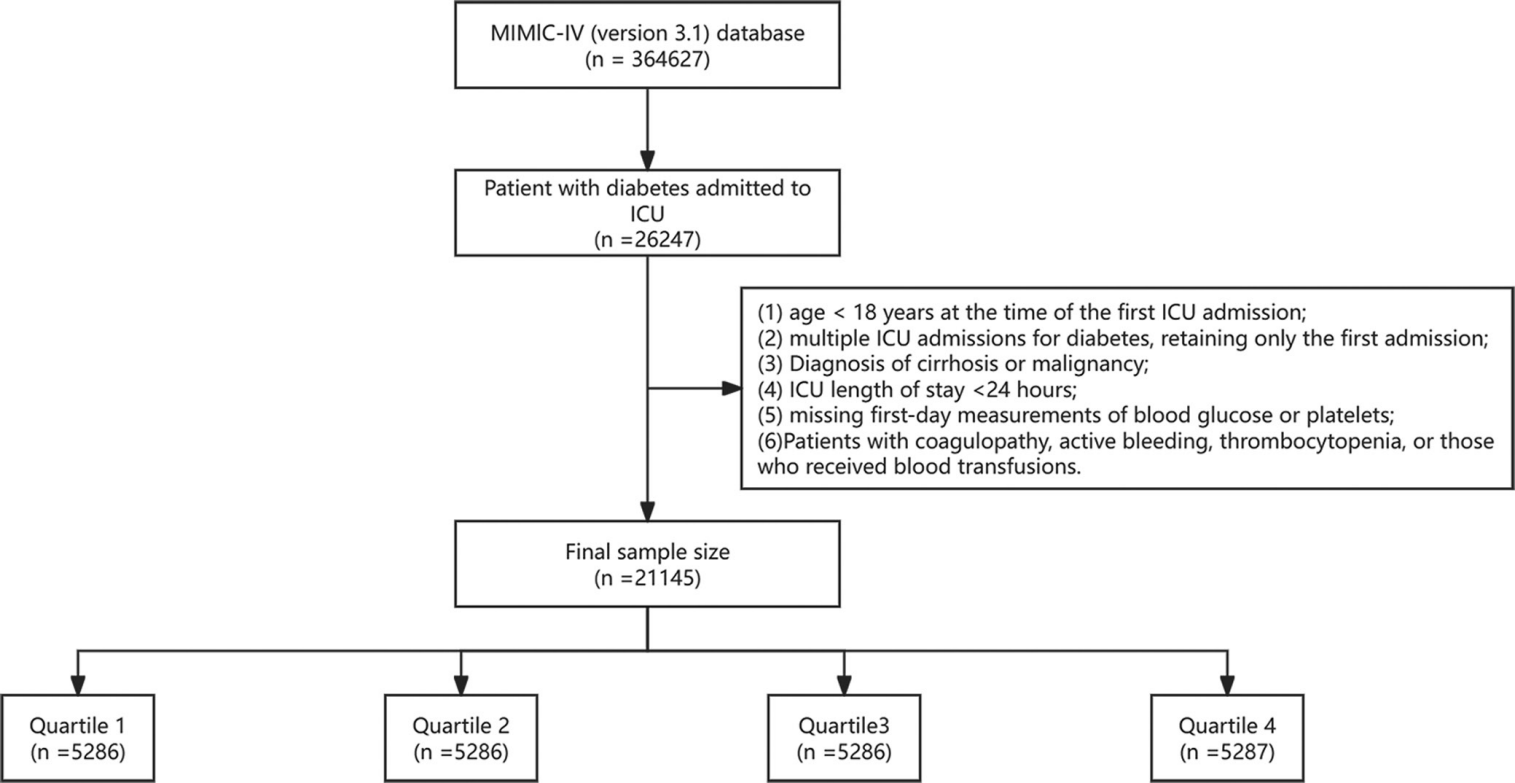
Flowchart of patient selection process from the MIMIC-IV database. ICU = intensive care unit, MIMIC-IV = Medical Information Mart for Intensive Care IV.

We performed a retrospective analysis using the MIMIC-IV (version 3.1) database from the MIT Laboratory for Computational Physiology, containing de-identified, high-fidelity records of adult intensive care unit (ICU) admissions at Beth Israel Deaconess Medical Center.^[19]^ One author (Yinan Zhao) completed the credentialing process and extracted the data. Eligible cases included ICU patients diagnosed with diabetes, identified via Ninth or Tenth Revision (ICD-9/10) codes. A total of 26,247 diabetic patients were initially identified from the MIMIC-IV database. Exclusions were made for patients who met any of the following criteria: age < 18 years at the time of the first ICU admission; multiple ICU admissions for diabetes, retaining only the first admission; diagnosis of cirrhosis or malignancy; ICU length of stay < 24 hours; missing first-day measurements of blood glucose or platelets; and patients with coagulopathy, active bleeding, thrombocytopenia, or those who received blood transfusions (coagulopathy and active bleeding: patients with active bleeding or clinical coagulopathy (defined as an international normalized ratio [INR] > 1.5 or abnormal prothrombin time [PT]/partial thromboplastin time [PTT] values) were excluded from the analysis, as these conditions could lead to inaccurate platelet counts and subsequently skew the BPR values; thrombocytopenia: patients with platelet counts < 50 K/μL were excluded, as low platelet counts could confound the relationship between platelet function and glucose levels; blood transfusion: patients who received a blood transfusion within the first 24 hours of ICU admission were excluded from the analysis, as transfusions can affect platelet counts and may result in artificially inflated or deflated BPR values). After applying these exclusion criteria, the study included 21,145 individuals, who were divided into quartiles based on the BPR (Fig. 1). For the ICD-9/ICD-10 codes of diabetes and its main complications, see the Table S1, Supplemental Digital Content 1.

### 2.2. Illness severity scores

Severity of illness scores are commonly used in ICU settings to assess the degree of illness in patients and predict outcomes. These scores are intended to quantify the severity of physiological derangements and provide prognostic information, which can guide clinical decision-making. The following scores were used in this study: Acute Physiology Score III (APSIII), a composite score that accounts for 17 different physiological parameters to assess the severity of a patient’s condition at ICU admission. It is used to predict mortality based on physiological disturbance (range: 0–71, higher scores indicate more severe illness); Simplified Acute Physiology Score II (SAPS-II),^[20]^ a severity score based on 12 physiological variables and patient age. It is designed to predict short-term mortality in critically ill patients (range: 0–163, higher scores indicate worse outcomes); Acute Physiology and Chronic Health Evaluation II (APACHE II),^[21]^ a severity scoring system that uses 12 physiological parameters, age, and chronic health conditions to predict mortality (range: 0–71); Oxford Acute Severity of Illness Score (OASIS), a severity score based on 6 clinical variables, including age, blood pressure, and respiratory rate (range: 0–48, higher scores reflect more severe illness); Sepsis-related Organ Failure Assessment (SOFA),^[22,23]^ a score that assesses organ failure in 6 organ systems: respiratory, coagulation, liver, cardiovascular, renal, and neurological function (range: 0–24, higher scores indicate more severe organ dysfunction); Charlson Comorbidity Index (CCI),^[24]^ a widely used method for assessing comorbidities based on the presence of various diseases, each weighted according to its potential impact on mortality. It is a prognostic tool for predicting long-term mortality and is often used to adjust for comorbidities in observational studies. Since diabetes is included as a component in the CCI, we accounted for its influence on the total comorbidity burden in the analysis. All patients in this research cohort are diabetes patients, and the original CCI contains diabetes entries. In order to avoid repeated assignment and accurately assess the burden of complications other than diabetes, this study excluded diabetes entries and recalculated the CCI score. The assignment standard refers to Charlson’s original literature. A higher score represents a heavier burden of complications. In this study, the CCI was included to account for the overall comorbidity burden and its potential impact on patient outcomes, independent of the direct influence of diabetes. By incorporating CCI, we could better assess the contribution of other comorbidities (such as cardiovascular disease, chronic kidney disease, and stroke) in the overall mortality risk, providing a more comprehensive view of the factors affecting survival.

### 2.3. Data collection

Data were queried from MIMIC-IV (MIT Laboratory for Computational Physiology, Massachusetts Institute of Technology) database using Structured Query Language with Python (version 3.10.6; Python Software Foundation). Four categories of variables documented within the first 24 hours of ICU admission were collected: demographics, including age, weight and sex. comorbidities, such as heart failure (HF), respiratory failure, atrial fibrillation (AF), acute kidney injury (AKI), cerebrovascular accident, hypertension, hyperlipoproteinemia, and sepsis. laboratory indicators, including red blood cells (RBC), white blood cells (WBC), platelet, hemoglobin, Hematocrit, red cell distribution width (RDW), blood glucose, sodium, total calcium, potassium, chloride, anion gap, creatinine, urea nitrogen, and coagulation parameters (PT, PTT and INR). severity of illness scores at admission, such as the APSIII, SAPS-II, APACHE II, OASIS, CCI, and SOFA. The BPR was calculated as the ratio of blood glucose-to-platelets. The follow-up period started at ICU admission and concluded at either discharge or in-hospital death.

Variables (such as absolute lymphocyte count, absolute neutrophil count and albumin) with over 20% missing data were excluded from the analysis. For variables with missing data of 20% or less, multiple imputation using the random forest method was applied via the “mice” package in R (refer to Table S2, Supplemental Digital Content 2).^[25,26]^ This approach allowed for the estimation of missing values based on the observed relationships between variables in the dataset, thereby preserving the overall data structure and minimizing bias.

### 2.4. Clinical outcomes

The primary outcomes were all-cause mortality during hospitalization, assessed at 2 time points: 28 days and 360 days after ICU admission.

### 2.5. Statistical analysis

Continuous variables were expressed as mean ± standard deviation or median (Interquartile range [IQR]), depending on distribution, which was assessed using the Kolmogorov–Smirnov test. For between-group comparisons, the *t*-test or 2-way analysis of variance (ANOVA) was applied to normally distributed data, while the Mann–Whitney *U* or Kruskal–Wallis tests were used for non-normal distributed data. Categorical variables are presented as counts (percentages). Kaplan–Meier survival curves were used to visualize the cumulative mortality rates for each quartile of the BPR at 28 days and 360 days. These curves were unadjusted, as we did not use adjusted Kaplan–Meier curves in this analysis. This approach allowed us to assess the overall trends without adjusting for covariates.

This study did not use binary logistic regression. The logistic regression mentioned in the previous communication was a preliminary exploratory analysis and was not included in the final text; The main outcome is time event data, so Cox proportional hazards model is used as the core analysis method, which is more suitable for survival data analysis. The selection criteria for covariates in multivariate models are based on clinical significance, single factor analysis with *P* < .05 variables, and exclusion of multicollinearity through GVIFs test. Subsequently, we applied Cox proportional hazards models to calculate hazard ratios (HRs) and 95% confidence intervals (CIs) for the relationship between BPR and mortality. Three prespecified models were used: model 1 (unadjusted), model 2 (adjusted for age, sex, and weight), model 3 (adjusted for age, sex, weight, HF, respiratory failure, AKI, sepsis, WBC, RBC, sodium, calcium total, potassium, chloride, anion gap, creatinine, and urea nitrogen). Potential multicollinearity among covariates was assessed using generalized variance inflation factors (GVIFs). Covariates with GVIFs greater than 10 were excluded from the final model to minimize multicollinearity, ensuring the robustness of the estimates (see Table S3, Supplemental Digital Content 3). Finally, the proportional hazards assumption for the Cox model was tested. If the assumption was violated, alternatives such as time-varying coefficients or stratified Cox models would have been considered, though no violations were detected in this analysis (see Fig. S1, Supplemental Digital Content 4). This study included weight, which can affect blood glucose metabolism, platelet levels, and severe prognosis; The cutoff value of 65 kg is based on previous studies on weight stratification of patients with severe diabetes, and it is the commonly used weight grouping threshold in clinical practice.

To investigate nonlinearity, baseline BPR was modeled using restricted cubic splines (4 knots) for both 28-day and 360-day hospital mortality. Receiver operating characteristic analysis was used to determine the optimal BPR cutoff. BPR entered the models either as a continuous variable or as an ordinal variable (quartiles; lowest quartile as the reference). The *P*-value for trend was calculated across quartiles.

We conducted prespecified subgroup analyses considering sex, age (≤65 vs >65 years), weight (≤65 vs >65 kg), sepsis, respiratory failure, AKI, and HF. Interactions between BPR and each stratifying variable were assessed using likelihood ratio tests. Statistical significance was determined by a 2-sided *P*-value of less than .05. The analyses utilized Python 3.10.6 and SPSS 26.0 (IBM, Armonk).

## 3. Results

A total of 21,145 critically ill patients with diabetes were included in the analysis. The median age was 69 years (IQR, 60–78), and 12,350 (58.41%) were male. The median BPR was 0.82 (IQR, 0.57–1.25). The 28-day hospital mortality rate was 10.83%, and the 360-day hospital mortality rate was 15.57% (see Table 1).

**Table 1:** Characteristics and outcomes of participants categorized by blood glucose-to-platelet ratio (BPR*).

Categories	Overall (N = 21145)	Q1 (N = 5286)	Q2 (N = 5286)	Q3 (N = 5286)	Q4 (N = 5287)	*P*-value
Age (yrs)	69 (60–78)	69 (60–78)	70 (61–78)	70 (61–78)	68 (59–77)	<.001
Weight (kg)	84.2 (70.1–100.4)	83.5 (69.0–100.9)	84.8 (71.3–101.2)	86.1 (72.0–101.2)	83.0 (70.0–99.1)	<.001
Sex: male	12,350 (58.41)	2791 (52.80)	2993 (56.62)	3238 (61.26)	3328 (62.95)	<.001
SOFA	4 (2–7)	4 (2–6)	4 (2–6)	4 (2–7)	6 (4–9)	<.001
APS III	44 (34–58)	43 (33–55)	42 (32–55)	42 (32–55)	50 (39–65)	<.001
SAPS II	36 (28–45)	35 (27–43)	35 (28–44)	35 (28–44)	39 (31–49)	<.001
OASIS	30 (25–37)	30 (25–36)	30 (25–36)	30 (25–36)	31 (26–38)	<.001
APACHE II	18 (13–23)	17 (13–22)	17 (13–22)	17 (13–22)	20 (15–25)	<.001
Commorbidities						
Heart failure	8277 (39.14)	2187 (41.37)	2074 (39.24)	2005 (37.93)	2011 (38.04)	<.001
Respiratory failure	6550 (30.98)	1645 (31.12)	1486 (28.11)	1521 (28.77)	1898 (35.90)	<.001
Atrial fibrillation	6997 (33.09)	1756 (33.22)	1810 (34.24)	1798 (34.01)	1633 (30.89)	<.001
Acute kidney injury	8798 (41.61)	2052 (38.82)	1908 (36.10)	2022 (38.25)	2816 (53.26)	<.001
Cerebrovascular accident	2299 (10.87)	557 (10.54)	594 (11.24)	603 (11.41)	545 (10.31)	.2
Hypertension	9037 (42.74)	2348 (44.42)	2401 (45.42)	2326 (44.00)	1962 (37.11)	<.001
Hyperlipoproteinemia	11300 (53.44)	2725 (51.55)	3001 (56.77)	3017 (57.08)	2557 (48.36)	<.001
Sepsis	3674 (17.38)	958 (18.12)	779 (14.74)	794 (15.02)	1143 (21.62)	<.001
CCI	6 (5–8)	6 (4–8)	6 (4–8)	6 (4–8)	7 (5–9)	<.001
Vital signs						
Heart rate (bpm)	86 (75–100)	86 (75–100)	84 (74–97)	84 (74–98)	89 (77–104)	<.001
Respiratory rate	18 (15–22)	19 (15–23)	18 (15–22)	18 (15–22)	19 (16–23)	<.001
SpO_2_	98 (95–100)	98 (95–100)	98 (96–100)	98 (96–100)	98 (95–100)	<.001
Laboratory tests						
WBC (K/μL)	10.7 (7.7–14.8)	11.3 (8.3–15.7)	10.8 (8.0–14.9)	10.7 (7.9–14.3)	9.8 (6.7–14.0)	<.001
RBC (m/μL)	3.46 (2.94–4.03)	3.48 (3.00–4.02)	3.50 (2.99–4.07)	3.49 (2.99–4.06)	3.36 (2.80–3.96)	<.001
Platelet (K/μL)	193 (140–258)	278 (224–353)	206 (168–255)	168 (134–216)	123 (83–171)	<.001
Hemoglobin (g/dL)	10.1 (8.6–11.7)	9.9 (8.6–11.4)	10.2 (8.7–11.8)	10.3 (8.8–12.0)	10.0 (8.5–11.7)	<.001
Hematocrit (%)	31.1 (26.8–36.0)	30.8 (26.9–35.3)	31.4 (27.0–36.2)	31.7 (27.2–36.6)	30.7 (26.0–35.7)	<.001
RDW (%)	14.7 (13.6–16.4)	15.1 (13.9–16.8)	14.6 (13.5–16.2)	14.4 (13.3–15.9)	14.9 (13.7–16.8)	<.001
Blood glucose (mg/dL)	152 (117–211)	113 (92–139)	140 (115–175)	169 (133–218)	232 (170–315)	<.001
BPR	0.82 (0.57–1.25)	0.43 (0.34–0.50)	0.69 (0.63–0.75)	1.00 (0.91–1.12)	1.79 (1.45–2.52)	
Sodium (mEq/L)	138 (135–141)	139 (135–141)	139 (136–141)	138 (136–141)	137 (134–140)	<.001
Total calcium (mg/dL)	8.4 (8.0–8.9)	8.5 (8.0–9.0)	8.5 (8.0–8.9)	8.4 (8.0–8.9)	8.4 (7.8–8.9)	<.001
Potassium (mEq/L)	4.20 (3.80–4.70)	4.20 (3.80–4.60)	4.20 (3.80–4.60)	4.30 (3.90–4.70)	4.30 (3.90–4.90)	<.001
Chloride (mEq/L)	104 (99–108)	103 (99–107)	104 (100–108)	104 (100–108)	103 (98–107)	<.001
Anion gap (mEq/L)	14 (12–17)	14 (12–17)	14 (11–16)	14 (11–17)	15 (13–19)	<.001
Creatinine (mg/dL)	1.1 (0.8–1.9)	1.1 (0.8–1.9)	1.1 (0.8–1.7)	1.1 (0.8–1.7)	1.4 (0.9–2.2)	<.001
Urea nitrogen (mg/dL)	24 (15–40)	23 (15–40)	22 (15–36)	22 (15–37)	29 (18–48)	<.001
PT (s)	14.2 (12.5–16.9)	14.0 (12.5–16.7)	14.0 (12.5–16.3)	14.1 (12.5–16.6)	14.6 (12.6–18.2)	<.001
PTT (s)	30.7 (27.1–37.1)	30.9 (27.4–36.7)	30.2 (26.9–35.9)	30.30 (26.8–36.2)	31.60 (27.4–39.7)	<.001
INR(PT)	1.3 (1.1–1.5)	1.3 (1.1–1.5)	1.3 (1.1–1.5)	1.3 (1.1–1.5)	1.3 (1.1–1.7)	<.001
Outcomes						
28-day Hospital mortality	2291 (10.83)	495 (9.36)	484 (9.16)	504 (9.53)	808 (15.28)	<.001
360-day Hospital mortality	3293 (15.57)	780 (14.76)	688 (13.02)	706 (13.36)	1119 (21.17)	<.001

APACHEII = acute physiology and chronic health evaluation II, APSIII = acute physiology score III, BPR = blood glucose-to-platelet ratio, CCI = charlson comorbidity index, INR = international normalized ratio, OASIS = oxford acute severity of illness score, PT = prothrombin time, PTT = partial thromboplastin time, RBC = red blood cell, RDW = red cell distribution width, SAPSII = Simplifed Acute Physiological Score II, SOFA = sequential organ failure assessment, SpO_2_ = O_2_ saturation pluseoxymetry, WBC = white blood cell.

* BPR: Q1 (0.07–0.57), Q2 (0.57–0.82), Q3 (0.82–1.25), Q4 (1.25–9.97).

### 3.1. Baseline characteristics

Table 1 provides a summary of baseline characteristics categorized by admission BPR quartiles. Patients were grouped into BPR quartiles as follows: Q1 (0.07–0.57), Q2 (0.57–0.82), Q3 (0.82–1.25), and Q4 (1.25–9.97). The median BPR values for these quartiles were 0.43 (IQR, 0.34–0.50), 0.69 (IQR, 0.63–0.75), 1.00 (IQR, 0.91–1.12), and 1.79 (IQR, 1.45–2.52), respectively. Compared with lower quartiles, Q4 patients had higher blood glucose levels, greater illness severity at admission, and a higher prevalence of hypertension, hyperlipoproteinemia, and sepsis. Additionally, Q4 patients had higher urea nitrogen levels. The outcomes showed that mortality rates increased across quartiles, although Q2 had slightly lower mortality compared to Q3. Specifically, 28-day hospital mortality rates were 9.36%, 9.16%, 9.53%, and 15.28% (*P* < .001), while 360-day hospital mortality rates were 14.76%, 13.02%, 13.36%, and 21.17% (*P* < .001) from Q1 to Q4. These findings indicate a strong positive association between higher BPR and increased all-cause mortality.

Table 2 displays baseline differences based on 360-day survival status. Non-survivors had higher severity scores, a greater prevalence of AKI and sepsis, and higher levels of RDW, blood glucose, anion gap, creatinine, urea nitrogen, PT, and PTT. Non-survivors also exhibited a higher BPR compared to survivors (0.92 vs 0.81, *P* < .001).

**Table 2:** Baseline characteristics of the survivors and non-survivors groups.

Categories	Overall (N = 21145)	Survivor (N = 17852)	Non-survivor (N = 3293)	*P*-value
Age (yr)	69 (60–78)	68 (59–77)	73 (64–81)	<.001
Weight (kg)	84.20 (70.50–100.43)	85.10 (71.20–101.50)	79.80 (67.50–95.30)	<.001
Sex: male	12,350 (58.41)	10,468 (58.64)	1882 (57.15)	.112
SOFA	4 (2–7)	4 (2–6)	7 (4–10)	<.001
APS III	44 (34–58)	42 (32–54)	58 (46–75)	<.001
SAPS II	36 (28–45)	35 (27–43)	46 (37–56)	<.001
OASIS	30 (25–37)	30 (24–35)	36 (29–42)	<.001
APACHE II	18 (13–23)	17 (13–22)	22 (18–28)	<.001
Commorbidities				
Heart failure	8277 (39.14)	6612 (37.04)	1665 (50.56)	<.001
Respiratory failure	6550 (30.98)	4665 (26.13)	1885 (57.24)	<.001
Atrial fibrillation	6997 (33.09)	5619 (31.48)	1378 (41.85)	<.001
Acute kidney injury	8798 (41.61)	6761 (37.87)	2037 (61.86)	<.001
Hypertension	9037 (42.74)	8008 (44.86)	1029 (31.25)	<.001
Hyperlipoproteinemia	11300 (53.44)	9785 (54.81)	1515 (46.01)	<.001
Sepsis	3674 (17.38)	2410 (13.50)	1264 (38.38)	<.001
CCI	6 (5–8)	6 (4–8)	8 (6–10)	<.001
Vital signs				
Heart rate (bpm)	86 (75–100)	85 (75–99)	90 (76–105)	<.001
Respiratory rate	18 (15–22)	18 (15–22)	20 (16–24)	<.001
SpO_2_	98 (95–100)	98 (96–100)	98 (95–100)	<.001
Laboratory tests				
WBC (K/μL)	10.7 (7.7–14.8)	10.6 (7.7–14.5)	11.5 (7.9–16.7)	<.001
RBC (m/μL)	3.46 (2.94–4.03)	3.48 (2.98–4.05)	3.32 (2.82–3.94)	<.001
Platelet (K/μL)	193 (140–258)	193 (142–256)	190 (123–265)	.047
Hemoglobin (g/dL)	10.1 (8.6–11.7)	10.2 (8.7–11.8)	9.7 (8.3–11.4)	<.001
Hematocrit (%)	31.1 (26.8–36.0)	31.2 (26.9–36.1)	30.6 (26.1–35.4)	<.001
RDW (%)	14.7 (13.6–16.4)	14.6 (13.5–16.1)	15.9 (14.5–18.1)	<.001
Blood glucose (mg/dL)	152 (117–211)	150 (116–207)	165 (121–229)	<.001
BPR	0.82 (0.57–1.25)	0.81 (0.56–1.21)	0.92 (0.58–1.54)	<.001
Sodium (mEq/L)	138 (135–141)	138 (135–141)	138 (134–141)	<.001
Total calcium (mg/dL)	8.4 (8.0–8.9)	8.5 (8.0–8.9)	8.4 (7.9–8.9)	.001
Potassium (mEq/L)	4.2 (3.8–4.7)	4.2 (3.8–4.7)	4.3 (3.9–4.9)	<.001
Chloride (mEq/L)	104 (99–108)	104 (99–108)	102 (97–107)	<.001
Anion gap (mEq/L)	14 (12–17)	14 (12–17)	16 (13–19)	<.001
Creatinine (mg/dL)	1.1 (0.8–1.9)	1.1 (0.8–1.8)	1.6 (1.0–2.9)	<.001
Urea nitrogen (mg/dL)	24 (15–40)	22 (15–37)	35 (22–56)	<.001
PT (s)	14.2 (12.5–16.9)	14.0 (12.4–16.5)	15.3 (13.2–20.0)	<.001
PTT (s)	30.7 (27.1–37.1)	30.4 (26.9–36.1)	33.3 (28.4–44.2)	<.001
INR(PT)	1.3 (1.1–1.5)	1.3 (1.1–1.5)	1.4 (1.2–1.8)	<.001

APACHEII = acute physiology and chronic health evaluation II, APSIII = acute physiology score III, BPR = blood glucose-to-platelet ratio, CCI = charlson comorbidity index, INR = international normalized ratio, OASIS = oxford acute severity of illness score, PT = prothrombin time, PTT = partial thromboplastin time, RBC = red blood cell, RDW = red cell distribution width, SAPSII = Simplifed Acute Physiological Score II, SOFA = sequential organ failure assessment, SpO_2_ = O_2_ saturation pluseoxymetry, WBC = white blood cell.

### 3.2. Primary outcomes

**Figure 2.**
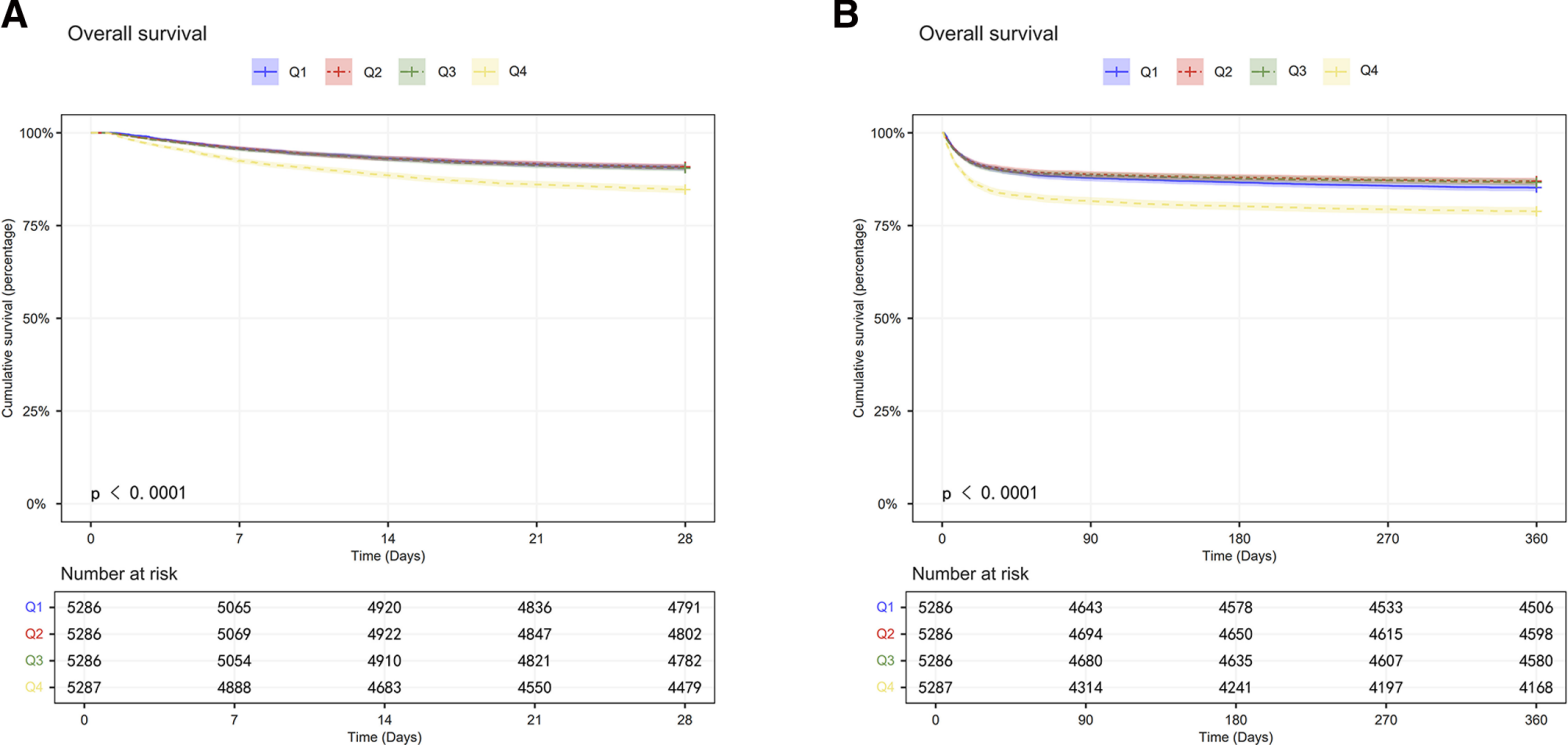
Kaplan–Meier curves displaying the cumulative chance of all-cause mortality by group at 28 days (A) and 360 days (B).

Kaplan–Meier curves by BPR quartile (Fig. 2) showed progressively lower survival rates for higher BPR groups, with both 28-day and 360-day hospital mortality significantly differing across quartiles (both *P* < .001).

Univariate Cox analyses (Table S4, Supplemental Digital Content 5) revealed that age, weight, sex, illness severity scores, comorbidities, and vital signs significantly predicted the 28-day hospital mortality rate (*P* < .05). Laboratory variables such as WBC, RBC, platelet count, hemoglobin, RDW, blood glucose, BPR, total calcium, potassium, chloride, anion gap, creatinine, urea nitrogen, PT, PTT, and INR were also individually associated (all *P* < .05).

In Cox proportional hazards models evaluating BPR, increased BPR as a continuous variable was associated with higher hospital mortality across all models: unadjusted (hazard ratio [HR] 1.03, 95% CI 1.02–1.04, *P* < .001), partially adjusted (HR 1.04, 95% CI 1.04–1.05, *P* < .001), and fully adjusted (HR 1.03, 95% CI 1.02–1.04, *P* < .001). When BPR was considered as a categorical exposure, patients in Q4 exhibited a higher 28-day hospital mortality risk compared to those in Q1 across all models: unadjusted HR 1.69 (95% CI 1.51–1.89, *P* < .001), partially adjusted HR 1.75 (1.56–1.96, *P* < .001), and fully adjusted HR 1.36 (1.22–1.53, *P* < .001; Table 3). Comparable trends were observed for 360-day mortality (Table 3).

**Table 3:** Cox proportional hazard ratios (HR) for 28-day and 360-day hospital mortality.

Categories	Model1			Model2			Model3		
	HR (95% CI)	*P*-value	*P* for trend	HR (95% CI)	*P*-value	*P* for trend	HR (95% CI)	*P*-value	*P* for trend
28-day hospital mortality									
Continuous variable per unit	1.03 (1.02–1.04)	<.001		1.04 (1.04–1.05)	<.001		1.03 (1.02–1.04)	<.001	
Quartile*			<.001			<.001			<.001
Q1 (N = 5286)	Reference								
Q2 (N = 5286)	0.98 (0.86–1.11)	.733		0.97 (0.85–1.10)	.616		1.07 (0.94–1.21)	.296	
Q3 (N = 5286)	1.02 (0.90–1.16)	.743		1.02 (0.90–1.16)	.722		1.06 (0.93–1.20)	.378	
Q4 (N = 5287)	1.69 (1.51–1.89)	<.001		1.75 (1.56–1.96)	<.001		1.36 (1.22–1.53)	<.001	
360-day hospital mortality									
Continuous variable per unit	1.03 (1.03–1.04)	<.001		1.04 (1.03–1.05)	<.001		1.03 (1.02–1.04)	<.001	
Quartile*			<.001			<.001			<.001
Q1 (N = 5286)	Reference								
Q2 (N = 5286)	0.88 (0.79–0.97)	.014		0.87 (0.79–0.97)	.009		0.97 (0.87–1.07)	.525	
Q3 (N = 5286)	0.90 (0.82–1.00)	.054		0.91 (0.82–1.00)	.057		0.96 (0.86–1.06)	.389	
Q4 (N = 5287)	1.50 (1.37–1.65)	<.001		1.54 (1.40–1.68)	<.001		1.24 (1.13–1.37)	<.001	

The analysis of 28-day and 360-day mortality rates is based on the same baseline cohort (n = 21145); Patients who die within 28 days are included in the 360-day mortality analysis and counted as events. The survival time is calculated based on the actual time of death, which conforms to the principle of whole team follow-up in survival analysis.

Final sample sizes after exclusions and imputation: 21,145 for all models.

Model 1: unadjusted.

Model 2: adjusted for age, sex, weight.

Model 3: adjusted for age, sex, weight, heart failure, respiratory failure, acute kidney injury, sepsis, white blood cell, red blood cell, sodium, total calcium, potassium, chloride, anion gap, creatinine, urea nitrogen.

* BPR: Q1 (0.07–0.57), Q2 (0.57–0.82), Q3 (0.82–1.25), Q4 (1.25–9.97).

**Figure 3.**
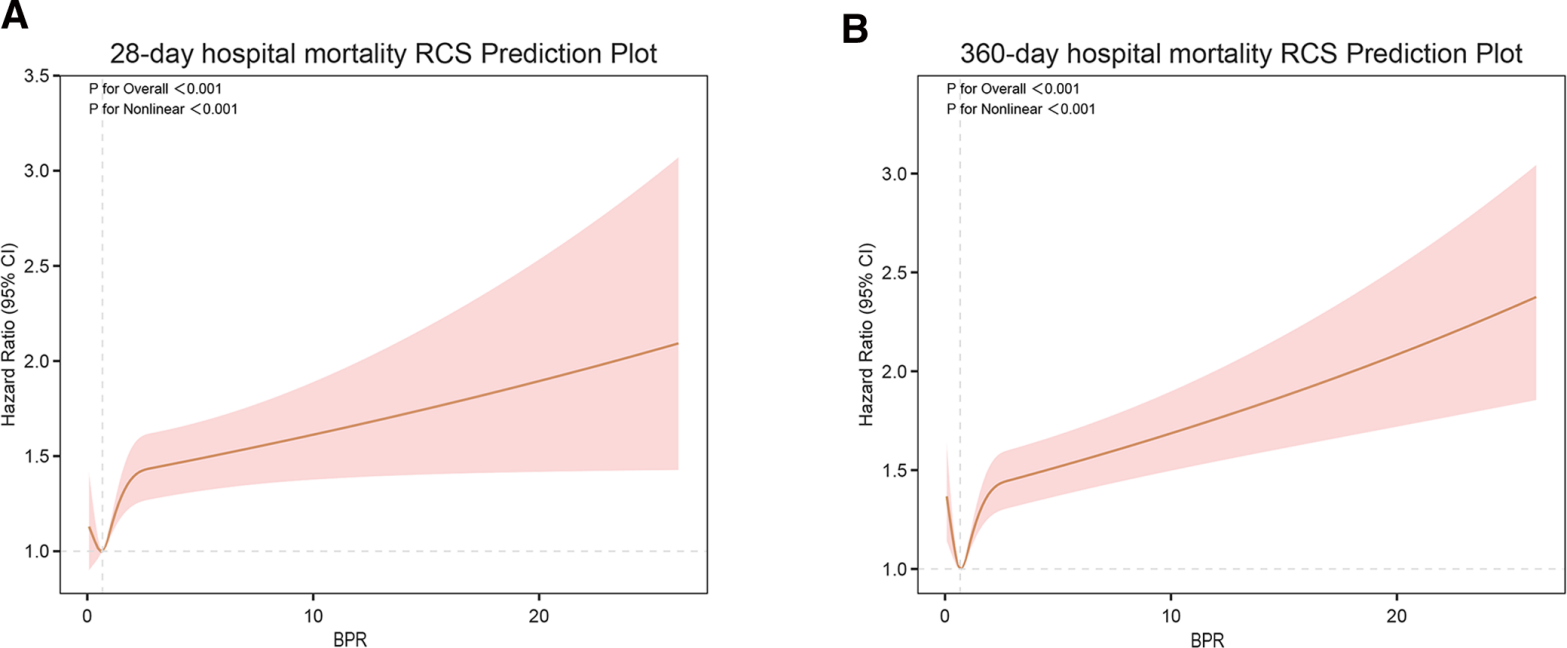
(A) RCS for 28-day hospital mortality. (B) RCS for 360-day hospital mortality. BPR = blood glucose/platelet ratio, CI = confidence interval, HR = hazard ratio, RCS = restricted cubic spline.

Restricted cubic spline analyses further characterized a dose–response relationship (Fig. 3A, B). As BPR increased, the predicted 28-day and 360-day hospital mortality rates increased approximately linearly. Tests for nonlinearity were significant for both outcomes (*P* < .001 for each).

### 3.3. Subgroup analysis

**Figure 4.**
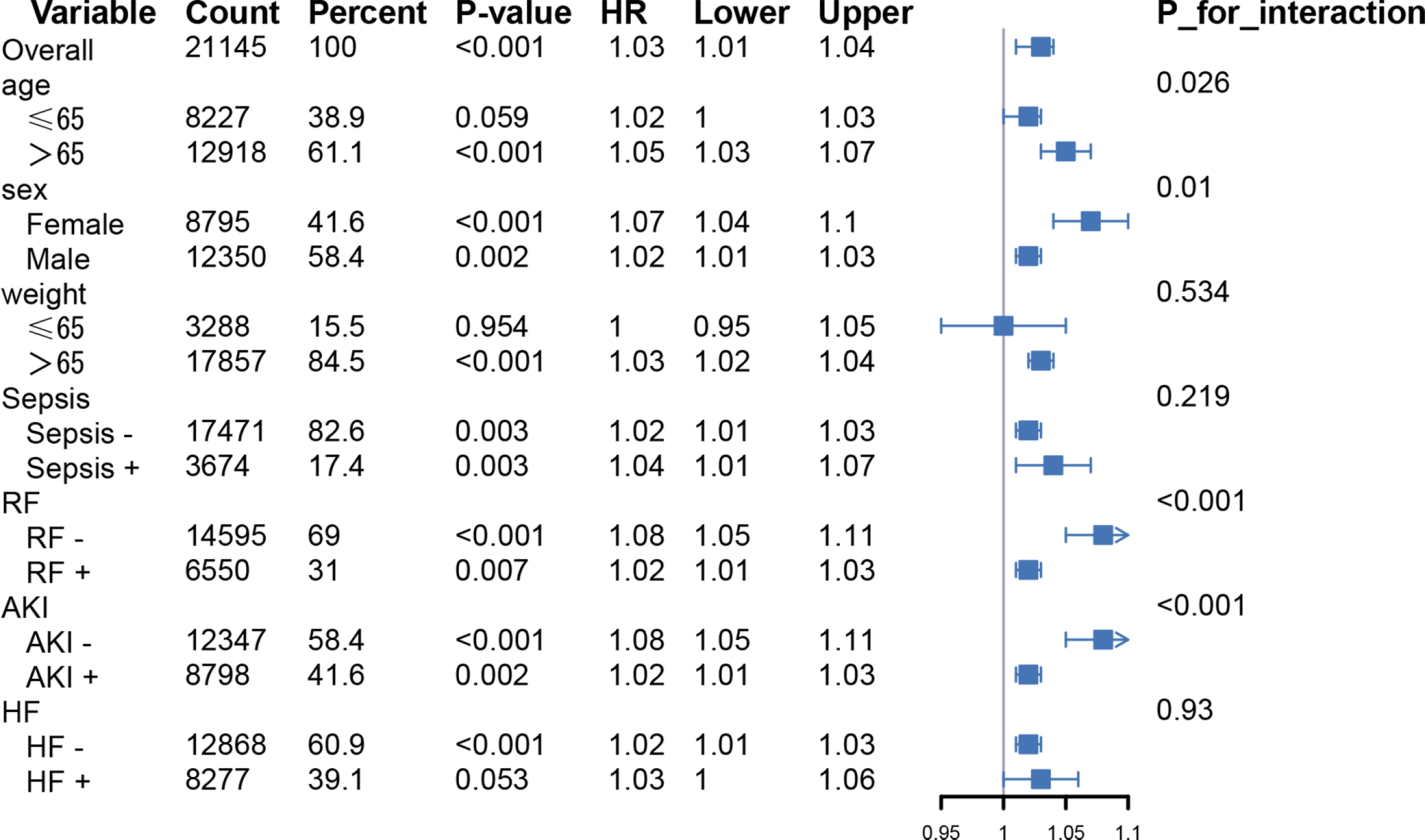
Forest plots of hazard ratios for the 28-day hospital mortality in different subgroups. It should be noted that the subgroup analyses were performed using adjusted models, accounting for the relevant covariates as outlined in the methods section. AF = atrial fibrillation, AKI = acute kidney injury, CI = confidence interval, HF = heart failure, HR = hazard ratio.

**Figure 5.**
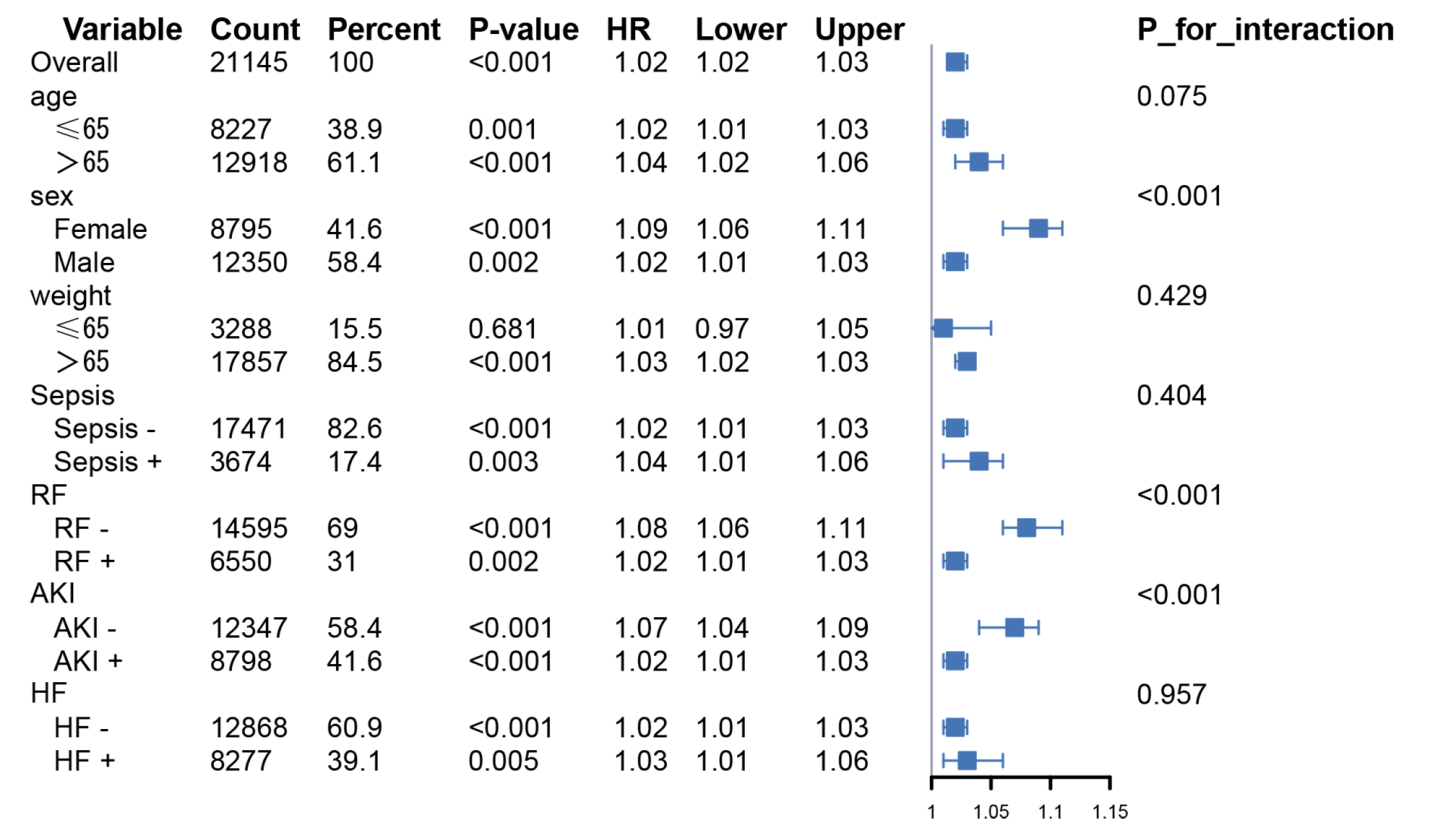
Forest plots of hazard ratios for the 360-day hospital mortality in different subgroups. It should be noted that the subgroup analyses were performed using adjusted models, accounting for the relevant covariates as outlined in the methods section. AF = atrial fibrillation, AKI = acute kidney injury, CI = confidence interval, HF = heart failure, HR = hazard ratio.

The associations between BPR and the primary endpoints were consistent across clinically relevant subgroups (Figs. 4 and 5). For 28-day hospital mortality, higher BPR as a continuous variable remained significantly associated with mortality in females (HR 1.07, 1.04–1.10), patients over the age of 65 years (HR 1.05, 1.03–1.07), those weighing over 65 kg (HR 1.03, 1.02–1.04), and patients without sepsis (HR 1.02, 1.01–1.03), without respiratory failure (HR 1.08, 1.05–1.11), without AKI (HR 1.08, 1.05–1.11), and without heart failure (HR 1.02, 1.01–1.03; Fig. 4). For 360-day hospital mortality, significant associations were seen in females (HR 1.09, 1.06–1.11) and males (HR 1.02, 1.01–1.03), in those aged > 65 years (HR 1.04, 1.02–1.06) and weighing > 65 kg (HR 1.03, 1.02–1.03), and among patients without sepsis (HR 1.02, 1.01–1.03), without respiratory failure (HR 1.08, 1.06–1.11), without AKI (HR 1.07, 1.04–1.09), and without heart failure (HR 1.02, 1.01–1.03; Fig. 5).

## 4. Discussion

In this study, we explored the relationship between the BPR and all-cause mortality in critically ill diabetic patients using data from the MIMIC-IV database. Our findings revealed a statistically significant association between higher BPR and increased hospital mortality, both at 28 days and 360 days. Specifically, higher BPR was linked to a higher risk of mortality in critically ill diabetic patients across different models. However, we did not adjust for illness severity in the final models, as severity scores were not included in these models. Thus, we must avoid suggesting that illness severity was accounted for in the adjusted analyses.

The relationship between blood glucose levels and mortality among diabetic patients has been extensively examined in the existing literature.^[12,13]^ Elevated glucose levels, a defining feature of diabetes, are acknowledged as a factor in a range of serious issues, including heart disease, kidney failure, and nerve disorders.^[9,10]^ These complications not only significantly diminish quality of life but are also directly correlated with increased mortality rates. Nevertheless, the involvement of platelets in the pathogenesis of these complications, particularly their influence on mortality, has been comparatively under-researched. In diabetic individuals, platelets frequently exhibit hyperactivity, facilitating thrombus formation and exacerbating cardiovascular events such as myocardial infarctions and strokes.^[14,27]^ This study elucidates the critical role of the BPR, an integrated marker of glycemic control and platelet activity, as a promising predictor of mortality risk in critically ill diabetic patients.

Our findings indicate that the BPR offers a more comprehensive evaluation of metabolic and thrombotic risks than glucose levels alone.^[28,29]^ This dual approach may be particularly significant for critically ill patients, where multiple factors contribute to mortality.^[30]^ While this study demonstrates an association, we do not assert predictive reliability or provide mechanistic confirmation of BPR’s role. Further studies are needed to confirm the causal nature of this relationship and determine the clinical utility of BPR in predicting mortality.

Our observations indicate that mortality risk exhibits a dose-dependent increase with elevated BPR values. The nonlinear relationship, as demonstrated by the restricted cubic spline analysis, reveals a pronounced escalation in mortality risk beyond a certain threshold. This finding underscores the critical importance of maintaining optimal blood glucose levels in conjunction with monitoring platelet activity in critically ill diabetic patients. Studies have shown that blood glucose control is closely related to the patient’s mortality rate.^[31]^ Although prior research has predominantly concentrated on glucose regulation as an isolated factor, our study proposes that a dual approach, incorporating platelet monitoring, may more comprehensively address the complex risks encountered by diabetic patients in intensive care environments.

Furthermore, the analysis of subgroup analyses revealed variations in the strength of association between BPR and mortality across different patient characteristics, including sex, age, and comorbidities. However, this should not be overstated as evidence of predictive power or effect modification, as we did not perform formal tests of effect modification or discrimination analyses. In fact, the modest per-unit effect observed in the continuous model, coupled with the stronger association confined to Q4, suggests possible nonlinearity or threshold effects, consistent with the findings from the restricted cubic spline analysis. Notably, in patients without AF, the BPR exhibited a stronger and more significant association with mortality. This finding suggests that certain underlying conditions, such as AF, may confound the relationship between metabolic dysregulation, platelet activity, and mortality. Related research has shown that AF itself is a standalone predictor of mortality.^[32,33]^ However, it is plausible that patients with AF are managed with medications or interventions, such as anticoagulants, which could alter their overall risk profile.^[34]^

This study provides a novel perspective on evaluating mortality risk in critically ill diabetic patients by examining the BPR. However, several limitations should be considered when interpreting our findings. As a retrospective analysis, this study cannot establish causality. While we made efforts to adjust for key confounders such as age, sex, weight, and comorbidities, there remains the possibility of residual confounding due to unmeasured variables like medication use, duration of diabetes, or specific causes of death, which could also influence mortality outcomes. Furthermore, since we assessed BPR only at baseline and did not account for its temporal changes during hospitalization, we cannot fully capture its dynamic role in predicting long-term mortality. Given that BPR may fluctuate during a patient’s ICU stay, future studies should examine how temporal variations in BPR impact long-term mortality and whether dynamic monitoring of this ratio could improve risk stratification over time.

Another limitation is the lack of sensitivity analyses, particularly excluding patients with AF or those without imputation. AF could potentially confound the relationship between metabolic dysregulation, platelet activity, and mortality, as patients with this condition may be treated differently, especially with anticoagulation therapies.^[35,36]^ Similarly, excluding patients with missing data or using different imputation methods might alter the findings. Without conducting such analyses, our results should be interpreted with caution, and further validation is needed to assess how these factors might influence the observed associations. Additionally, while the MIMIC-IV database provides a wealth of data from critically ill patients, it does not include certain variables, such as medication use, duration of diabetes, and causes of death, all of which could provide further insights into the mechanisms behind mortality outcomes in this population.

Finally, reverse causation remains a potential concern in this study. It is plausible that higher BPR levels could be a consequence of the severity of critical illness, rather than a predictor of mortality. This observation highlights the need for prospective studies that can better establish the directionality of the relationship between BPR and mortality. Moreover, the single-center nature of the MIMIC-IV database limits the generalizability of our findings. The data are derived from a single institution, which may not represent the broader population of critically ill diabetic patients. The findings should therefore be validated in diverse patient cohorts and across different healthcare settings to ensure their applicability to other hospitals or regions with distinct patient populations and treatment practices.

## 5. Conclusion

In conclusion, our study demonstrates a statistically significant association between higher BPR and increased all-cause mortality in critically ill diabetic patients. However, the small effect size and the lack of an intuitive clinical scale for BPR raise concerns about its practical application as a predictor of mortality. Although the findings suggest potential utility for BPR in risk stratification, its clinical relevance remains uncertain, and further research is needed to validate these findings in diverse clinical settings. Future studies should focus on determining the optimal threshold for BPR, exploring its role in dynamic monitoring, and assessing its potential to guide therapeutic interventions for high-risk diabetic patients.

## Acknowledgments

The authors sincerely thank the MIMIC official team for their outstanding contributions, which are of significant importance.

## Author contributions

**Conceptualization:** Yinan Zhao.

**Data curation:** Yinan Zhao.

**Formal analysis:** Yinan Zhao, Faying Li.

**Investigation:** Yinan Zhao.

**Methodology:** Yinan Zhao, Guoying Yu.

**Project administration:** Yinan Zhao.

**Resources:** Yinan Zhao, Guoying Yu.

**Software:** Yinan Zhao, Faying Li, Guoying Yu.

**Supervision:** Yinan Zhao.

**Validation:** Yinan Zhao, Faying Li.

**Visualization:** Yinan Zhao, Faying Li.

**Writing – original draft:** Yinan Zhao.

**Writing – review & editing:** Yinan Zhao.










