# Supplementary material for: Association of the blood glucose-to-platelet ratio with all-cause mortality in diabetic patients: An analysis of the MIMIC-IV database
Source: Medicine (Baltimore). 2026 Aug 7;105(32):e50128. doi: 10.1097/MD.0000000000050128 (PMC13456850; doi:10.1097/MD.0000000000050128)
Supplement: Supplementary file 1 [file medi-105-e50128-s001.docx]

E08, E080, E0801, E081, E0811, E082, E0829, E083, E0831, E08311, E0832, E08321, E083211, E083212, E083213, E083219, E08329, E083291, E083292, E083293, E083299, E0833, E08331, E083311, E083312, E0821, E083313, E083319, E08339, E083391, E083392, E083393, E083399, E0834, E08341, E083411, E083412, E083413, E083419, E08349, E083491, E083492, E083493, E083499, E0835, E08351, E083511, E083512, E083519, E08352, E083521, E083522, E0837X9, E083523, E083529, E08353, E083531, E083532, E083533, E083539, E08354, E083541, E083542, E083543, E083549, E08355, E083551, E083552, E11, E083553, E083559, E08359, E083591, E083592, E083593, E083599, E0836, E0837, E0837X1, E0837X2, E0837X3, E0839, E084, E0841, E0844, E0849, E085, E0852, E0859, E086, E0861, E08610, E08618, E0862, E08620, E08621, E08622, E08628, E0863, E08630, E08638, E0864, E08641, E088, E09, E090, E0901, E091, E0911, E092, E0929, E0851, E0869, E093, E0931, E09311, E0932, E09321, E093211, E093212, E093213, E093219, E09329, E093291, E093292, E093293, E0933, E09331, E093311, E093312, E093313, E093319, E09339, E093391, E093392, E093393, E093399, E0934, E09341, E093411, E093412, E09319, E093413, E093419, E09349, E093491, E093492, E093493, E093499, E0935, E09351, E093511, E093512, E093513, E093519, E09352, E093521, E093522, E093523, E093529, E09353, E093531, E093532, E093533, E093539, E13610, E09354, E093541, E0959, E093542, E093543, E093549, E09355, E093551, E093552, E093553, E093559, E09359, E093591, E093592, E093593, E093599, E0936, E0937, E0937X1, E0937X2, E0937X3, E0937X9, E0939, E094, E0941, E0944, E0949, E095, E0951, E0952, E096, E0961, E09610, E09618, E0962, E09620, E09622, E09628, E0963, E09630, E09638, E0964, E09641, E10, E101, E102, E103, E1031, E1032, E103211, E0969, E10321, E103212, E1033, E10331, E1034, E10341, E103412, E103419, E103499, E1035, E10352, E103521, E103529, E103311, E103312, E103313, E103319, E13618, E103391, E103392, E103399, E10353, E103533, E103539, E10354, E103541, E103542, E103543, E103549, E10355, E103551, E103552, E103553, E1037, E1037X1, E1037X2, E1037X3, E1037X9, E104, E105, E103532, E103591, E1041, E106, E1061, E1062, E1063, E10630, E10638, E1064, E110, E111, E1111, E112, E113, E1131, E1132, E11321, E113211, E1133, E1134, E113411, E113412, E1135, E11352, E113523, E113529, E11331, E113312, E113392, E113419, E11349, E113491, E11353, E113531, E113539, E11354, E113541, E113543, E113549, E11355, E113551, E113552, E113559, E1137, E1137X1, E1137X2, E1137X3, E114, E115, E113532, E113533, E113542, E1137X9, E116, E1161, E1162, E1163, E11630, E11638, E1164, E13, E130, E1301, E131, E132, E133, E1331, E13311, E1332, E13321, E133211, E133212, E133213, E133219, E13329, E133291, E11618, E13319, E133292, E133293, E133299, E1333, E13331, E133311, E133312, E133313, E133319, E13339, E133391, E133392, E133393, E133399, E1334, E13341, E133411, E133412, E133413, E133419, E13349, E133491, E133492, E133493, E133499, E1335, E13351, E133511, E133512, E133513, E133519, E13352, E133521, E133522, E133523, E133529, E13353, E133531, E133532, E133533, E133539, E13354, E133541, E133542, E133543, E133549, E13355, E133551, E133552, E133553, E133559, E13359, E133591, E133592, E133593, E133599, E1336, E1337, E1337X1, E1337X2, E1337X3, E1337X9, E1339, E134, E1341, E1344, E1349, E135, E1352, E136, E1361, E1362, E13620, E13628, E1363, E13630, E13638, E1364, E13641, E1343, E1351, E13621, E138, E710, E7411, O24, O240, O2401, O24019, O241, O2411, O24119, O243, O2431, O24312, O24319, O244, O2441, O24415, O2442, O2443, O24435, O248, O2481, O24811, O24812, O24813, O24819, O2482, O2483, O249, O2491, O24013, O24111, O24113, O2413, O24311, O24313, O2432, O2433, O24425, O24429, O24434, O24912, O24919, O2493, O24911, O24913, O2492, P700, P701, P702, V1221, Z131, Z7985, Z8631, 24900, 24901, 24911, 24920, 24940, 24941, 24950, 24951, 24960, 24961, 24980, 24981, 24990, 24991, 25000, 25002, 25012, 25020, 25022, 25030, 25032, 25040, 25042, 25050, 25052, 25060, 25062, 25070, 25072, 25080, 25082, 25090, 25092, 2714, 3572, 36201, 36202, 36203, 36204, 36205, 36206, 36207, 36641, 64801, 64803, 64804, 9623, E0800, E0810, E0822, E08319, E083513, E0840, E0842, E0843, E08649, E0865, E089, E0900, E0910, E0921, E0922, E093299, E0940, E0942, E0943, E09621, E09649, E0965, E098, E099, E1010, E1011, E1021, E1022, E1029, E10311, E10319, E103213, E103219, E10329, E103291, E103292, E103293, E103299, E10339, E103393, E103411, E103413, E10349, E103491, E103492, E103493, E10351, E103511, E103512, E103513, E103519, E103522, E103523, E103531, E103559, E10359, E103592, E103593, E103599, E1036, E1039, E1040, E1042, E1043, E1044, E1049, E1051, E1052, E1059, E10610, E10618, E10620, E10621, E10622, E10628, E10641, E10649, E1069, E108, E109, E1100, E1101, E1110, E1121, E1122, E1129, E11311, E11319, E113212, E113213, E113219, E11329, E113291, E113292, E113293, E113299, E113311, E113313, E11628, E113319, E11339, E113391, E113393, E113399, E11341, E113413, E113492, E113493, E113499, E11351, E113511, E113512, E113513, E113519, E113521, E113522, E113553, E11359, E113591, E113592, E113593, E113599, E1136, E1139, E1140, E1141, E1142, E1143, E1144, E1149, E1151, E1152, E1159, E11610, E11620, E11621, E11622, E11641, E11649, E1165, E1169, E118, E119, E1300, E1310, E1311, E1321, , E1329, E1340, E1342, E1359, E13622, E13649, E1365, E1369, E139, E9323, O24011, O24012, O2402, O2403, O24112, O2412, O24410, O24414, O24419, O24420, O24424, O24430, O24439, R7303, R81, V180, V771, Z833, Z8632, E1065, 24910, 24921, 24930, 24931, 24970, 24971, 25010, 64800, 64802
