# Supplementary material for: Association of the blood glucose-to-platelet ratio with all-cause mortality in diabetic patients: An analysis of the MIMIC-IV database
Source: Medicine (Baltimore). 2026 Aug 7;105(32):e50128. doi: 10.1097/MD.0000000000050128 (PMC13456850; doi:10.1097/MD.0000000000050128)
Supplement: Supplementary file 2 [file medi-105-e50128-s003.docx]

**Table S3.** Generalized variance inflation factors of covariates

| **Covariates** | **GVIF** |
| --- | --- |
| Age | 1.2103 |
| Sex | 1.0773 |
| Weight | 1.2019 |
| Heart failure | 1.1598 |
| Respiratory failure | 1.1552 |
| Acute kidney injury | 1.3062 |
| Sepsis | 1.2191 |
| White blood cell | 1.0560 |
| Red blood cell | 1.1404 |
| Sodium | 2.9089 |
| Total calcium | 1.2162 |
| Potassium | 1.2452 |
| Chloride | 3.3166 |
| Anion gap | 1.7364 |
| Creatinine | 2.1065 |
| Urea nitrogen | 2.0846 |
