# Supplementary material for: Association of the blood glucose-to-platelet ratio with all-cause mortality in diabetic patients: An analysis of the MIMIC-IV database
Source: Medicine (Baltimore). 2026 Aug 7;105(32):e50128. doi: 10.1097/MD.0000000000050128 (PMC13456850; doi:10.1097/MD.0000000000050128)
Supplement: Supplementary file 3 [file medi-105-e50128-s005.docx]

| Table S4. Univariate Cox Proportional Hazards Model Analysis | | | |
| --- | --- | --- | --- |
| **Categories** | **Overall (N=21145)** | **Hazard Ratio(HR)** | **P-value** |
| Age (years) | 68.200 (13.49) | 1.031 (1.028 - 1.035) | ＜0.001 |
| Weight (kg) | 87.930 (27.18) | 0.992 (0.990 - 0.994) | ＜0.001 |
| Sex: male | 12350 (58.40) | 0.910 (0.838 - 0.988) | 0.025 |
| SOFA | 5.020 (3.35) | 1.230 (1.218 - 1.242) | ＜0.001 |
| APS III | 47.73 (19.89) | 1.036 (1.034 - 1.037) | ＜0.001 |
| SAPS II | 37.61 (13.38) | 1.059 (1.056 - 1.062) | ＜0.001 |
| OASIS | 31.17 (8.49) | 1.094 (1.089 - 1.099) | ＜0.001 |
| APACHE II | 18.50 (7.15) | 1.105 (1.099 - 1.110) | ＜0.001 |
| Commorbidities |  |  |  |
| Heart failure | 8277 (39.1) | 1.485 (1.369 - 1.612) | ＜0.001 |
| Respiratory failure | 6550 (31.0) | 3.895 (3.581 - 4.238) | ＜0.001 |
| Atrial fibrillation | 6997 (33.1) | 1.489 (1.371 - 1.618) | ＜0.001 |
| Acute Kidney Injury | 8798 (41.6) | 2.683 (2.463 - 2.923) | ＜0.001 |
| Hypertension | 9037 (42.7) | 0.668 (0.612 - 0.728) | ＜0.001 |
| Hyperlipoproteinemia | 11300 (53.4) | 0.719 (0.663 - 0.781) | ＜0.001 |
| Sepsis | 3674 (17.4) | 3.668 (3.375 - 3.986) | ＜0.001 |
| CCI | 6.54 (2.84) | 1.208 (1.192 - 1.224) | ＜0.001 |
| Vital signs |  |  |  |
| Heart rate (bpm) | 88.18 (20.50) | 1.004 (1.004 - 1.005) | ＜0.001 |
| Respiratory rate | 19.08 (6.28) | 1.042 (1.037 - 1.047) | ＜0.001 |
| SpO_2_ | 97.06 (3.87) | 0.966 (0.960 - 0.972) | ＜0.001 |
| Laboratory tests |  |  |  |
| WBC (K/uL) | 12.20 (8.70) | 1.009 (1.007 - 1.011) | ＜0.001 |
| RBC (m/uL) | 3.52 (0.78) | 0.923 (0.875 - 0.974) | 0.003 |
| Platelet (K/uL) | 208.46 (104.84) | 1.000 (0.999 - 1.000) | 0.025 |
| Hemoglobin (g/dL) | 10.28 (2.20) | 0.976 (0.958 - 0.995) | 0.012 |
| Hematocrit (%) | 31.62 (6.55) | 1.003 (0.997 - 1.010) | 0.275 |
| RDW (%) | 15.30 (2.37) | 1.174 (1.159 - 1.190) | ＜0.001 |
| Blood glucose (mg/dL) | 178.82 (103.33) | 1.001 (1.001 - 1.001) | ＜0.001 |
| BPR | 1.15 (1.60) | 1.034 (1.026 - 1.041) | ＜0.001 |
| Sodium (mEq/L) | 137.97 (5.44) | 0.995 (0.987 - 1.002) | 0.172 |
| Calcium total (mg/dL) | 8.45 (0.84) | 0.889 (0.846 - 0.935) | ＜0.001 |
| Potassium (mEq/L) | 4.34 (0.78) | 1.201 (1.145 - 1.261) | ＜0.001 |
| Chloride (mEq/L) | 103.12 (6.95) | 0.973 (0.968 - 0.979) | ＜0.001 |
| Anion gap (mEq/L) | 14.90 (4.83) | 1.080 (1.073 - 1.087) | ＜0.001 |
| Creatinine (mg/dL) | 1.81 (1.89) | 1.091 (1.075 - 1.107) | ＜0.001 |
| Urea nitrogen (mg/dL) | 32.18 (25.38) | 1.013 (1.012 - 1.014) | ＜0.001 |
| PT (sec) | 16.64 (9.34) | 1.018 (1.016 - 1.021) | ＜0.001 |
| PTT (sec) | 37.99 (23.83) | 1.009 (1.008 - 1.010) | ＜0.001 |
| INR(PT) | 1.53 (0.90) | 1.211 (1.182 - 1.242) | ＜0.001 |
| Abbreviation: SOFA, sequential organ failure assessment; CCI, charlson comorbidity index; APSIII, acute physiology score III; SAPSII, simplifed acute physiological score II; OASIS, oxford acute severity of illness score; APACHEII, acute physiology and chronic health evaluation II; SpO2, O2 saturation pluseoxymetry; WBC, white blood cell; RBC, red blood cell; RDW, red cell distribution width; BPR, blood glucose/Platelet ratio, prothrombin time; PT, prothrombin time; PTT, partial thromboplastin time; INR, international normalized ratio | | | |
|  |  |  |  |
|  |  |  |  |
|  |  |  |  |
|  |  |  |  |
|  |  |  |  |
|  |  |  |  |
|  |  |  |  |
|  |  |  |  |
|  |  |  |  |
